# Cell-Free Genomic DNA Release into Serum of Wild Boar and Domestic Pigs Infected with Highly Virulent African Swine Fever Virus

**DOI:** 10.3390/pathogens14121228

**Published:** 2025-12-01

**Authors:** Ann Sofie Olesen, Louise Lohse, Graham J. Belsham

**Affiliations:** 1 Section for Veterinary Virology, Department of Virology and Microbiological Preparedness, Statens Serum Institut, Artillerivej 5, DK-2300 Copenhagen, Denmark; lolo@ssi.dk; 2 Section for Bacteria and Viruses, Department of Veterinary and Animal Sciences, University of Copenhagen, Stigbøjlen 4, DK-1870 Frederiksberg, Denmark; grbe@sund.ku.dk

**Keywords:** African swine fever virus, cell-damage, cell-free DNA, genomic DNA, lactate dehydrogenase, mitochondrial DNA

## Abstract

African swine fever virus (ASFV) is the cause of a severe hemorrhagic disease in domestic pigs and wild boar. Currently, a highly virulent genotype II ASFV is causing massive pig mortality worldwide. In its acute form, the disease is characterized by high fever, a range of non-specific clinical signs and cell death. In this study, we demonstrate a greatly elevated level (>1000-fold) of cell-free DNA (cfDNA), more specifically, fragmented host genomic DNA (gDNA), in serum from both wild boar and domestic pigs infected with a highly virulent genotype II ASFV. Increases were also observed, to a lesser extent, in the serum levels of mitochondrial DNA (between 4- to >500-fold). For comparison, release of the cytoplasmic enzyme, lactate dehydrogenase, which is a commonly used marker for cellular damage, was also found to be elevated in some animals, but with less consistency. These results indicate that gDNA in serum (i.e., cfDNA) can be a useful marker for cell death during infection with highly virulent variants of the virus, and could be a promising biomarker to elucidate the pathogenesis of ASFV infection in both domestic pigs and wild boar in future studies.

## 1. Introduction

African swine fever (ASF) is a severe hemorrhagic disease of domestic pigs and wild boar [1,2]. The disease is caused by African swine fever virus (ASFV), a large double-stranded DNA virus, the only member of the *Asfarviridae* family [3] and the only known DNA arbovirus [4]. Since the introduction of a highly virulent ASFV (of genotype II) from Africa into Georgia in 2007 [5,6], the distribution of the virus has reached pandemic proportions, with massive transmission within Europe and Asia and introduction into the Americas, resulting in huge losses of swine and major socioeconomic impact worldwide [7,8]. Infection with highly virulent ASFV often leads to a peracute to acute disease progression, with high case fatality in infected domestic pigs and wild boar. Clinical signs are usually nonspecific, with high fever, depression and reduced feed intake [9,10,11,12]. In its acute form, other clinical signs include skin hemorrhages, vomiting and bloody diarrhea. Furthermore, necropsy can reveal an enlarged, fragile spleen, enlarged hemorrhagic lymph nodes and internal bleeding. During peracute disease progression, death can occur without pronounced clinical signs and few pathological findings are sometimes reported [10,11,12]. In infected suids, the virus initially infects cells of the mononuclear phagocyte system, mainly monocytes and macrophages [13], and as the disease progresses severe dysregulation of the immune system (including a cytokine storm) and cell death occurs [14]. During infection with ASFV, cell death has been attributed to both apoptosis and necrosis [15,16].

Cell death (i.e., via apoptosis and necrosis) can lead to the release of fragmented, cellular genomic DNA (cell-free DNA, cfDNA) into the circulation, and this cfDNA has been described as a useful biomarker for cell damage [17]. The presence of these biomarkers can be readily detected in serum or plasma using real-time quantitative PCR (qPCR) assays with a small target size. We have previously shown that the production of cfDNA is an indicator of severe disease in domestic pigs that were infected with a highly virulent genotype II ASFV from Poland [18]. The aim of the current study was to investigate the presence of markers of cellular damage, including cfDNA, in the serum of domestic pigs and, additionally, of wild boar that had been experimentally infected with a highly virulent genotype II ASFV from Armenia, as recently described [19].

## 2. Materials and Methods

### 2.1. Serum Samples

The serum samples were kindly provided from a study previously performed at the Animal and Plant Health Agency (APHA) in the UK. [19]. No new animal experiments were performed for the analyses presented here. Briefly, for the animal experiment, domestic pigs and wild boar were inoculated intranasally with 10^4^ HAD_50_ of the highly virulent genotype II ASFV, Armenia 2007. In the current analysis, serum samples obtained from 16 domestic pigs and 16 wild boar were included (see Supplementary Table S1). Blood samples were obtained from all 32 animals before ASFV inoculation, and on days 1, 2, 3, 5 and 8 post infection (dpi), as well as postmortem (PM), from pigs randomly assigned to these sampling days prior to the experiment (for specific sampling days, see Supplementary Table S1). Following sampling, the serum samples were stored at −80 °C until further analysis. Separate results from this experiment have been published previously by Sánchez-Cordón et al. [19], but there is no overlap with the analyses performed on the serum samples described here.

### 2.2. Analysis for Host DNA in the Serum Samples

For the current study, nucleic acids were extracted from the serum samples using the MagNA Pure 96 system (Roche, Basel, Switzerland), as described previously [12]. Following purification, the samples were analyzed by qPCR using the CFX Opus Real-Time PCR System (Bio-Rad, Hercules, CA, USA). For all performed qPCR assays, a positive result was defined as a fluorescent emission signal appearing above background within 42 cycles (reported as Ct values). ASFV DNA was detected using the assay described by Tignon et al. [20], and absolute quantification performed as described previously [21], while genomic and mitochondrial DNA (mtDNA) (i.e., as used to determine the level of the *Sus scrofa* cytoskeletal β-actin gene and *Sus scrofa* mitochondrial cytochrome b gene) were assayed and absolute quantification (genome copies/mL) performed, as described by Olesen et al. [18], using the assays developed by Tignon et al. [20] and Forth [22], respectively.

In addition, an assay designed to distinguish between samples from domestic pigs and wild boar [23] was performed on selected samples (see Supplementary Table S1) to confirm the source of the serum samples that had been supplied [19] and to investigate if the level of this host gene sequence in serum was also affected during ASFV infection. The assay distinguishes between SNPs in the nuclear receptor subfamily 6 group A (*NR6A1*) gene, located on chromosome 1 [23].

### 2.3. Analysis for a Protein Marker of Cell Damage, Lactate Dehydrogenase, in the Serum Samples

The activity of lactate dehydrogenase (LDH) in serum samples was quantified using a commercial assay kit (Sigma-Aldrich, St. Louis, MO, USA, catalog number MAK066) according to the manufacturer’s instructions, as described previously [18]. In the assay, 1:10 dilutions of the serum in 1× Dulbecco’s phosphate-buffered saline (1× DPBS) (Gibco Thermo Fischer Scientific, Waltham, MA, USA) were used, together with a SunriseTM absorbance microplate reader (Tecan, Männedorf, Switzerland) for the measurements. Results were calculated according to the manufacturer’s instructions and are presented as milliUnits (mU)/mL.

### 2.4. Data Analyses

Production of graphs and data analyses, including calculations of the Spearman rank correlation coefficients that were used in order to compare non-normally distributed variables, were performed using GraphPad prism 9.0 (GraphPad Software, Boston, MA, USA). Standard curves for calculation of genome copy numbers were produced using Excel (LTSC, Microsoft, Redmond, WA, USA).

## 3. Results

**Figure 1:**
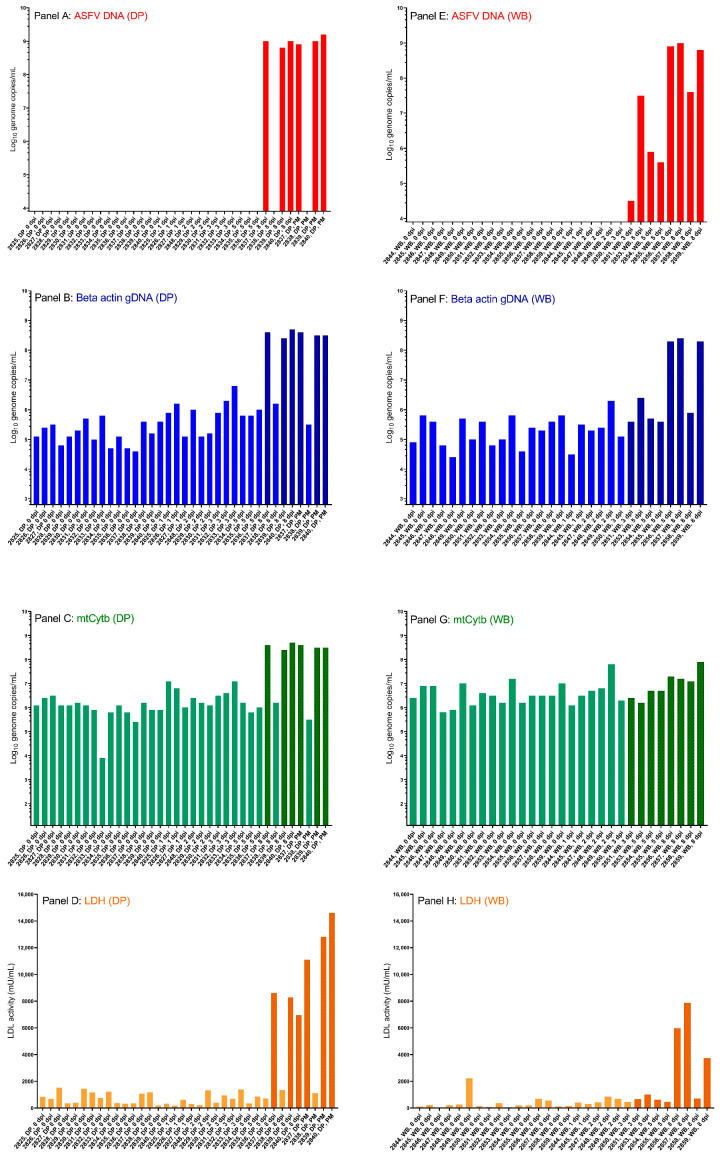
ASFV DNA, β-actin gDNA, mtDNA and LDH levels in domestic pig and wild boar serum. Nucleic acids isolated from serum samples were assayed by qPCR for the levels of ASFV DNA, β-actin gDNA and mtDNA, and absolute copy numbers/mL were determined from standard curves (panels (**A**–**C**) for domestic pigs and panels (**E**–**G**) for wild boar). The Ct values are shown in Supplementary Table S1. The serum samples were also assayed for the presence of the LDH enzyme, a marker for tissue damage (panel (**D**) for domestic pigs and panel (**H**) for wild boar). In panels (**B**–**D**,**F**–**H**) a darker color and shading of the column indicates that ASFV DNA was detected in serum from the pig or wild boar on that sampling day (also see panels (**A**,**E**). DP = domestic pig, WB = wild boar, PM = postmortem. Created using GraphPad prism 9.0 (GraphPad Software).

In this study, serum samples collected from domestic pigs and wild boar before and at different time points after infection with a highly virulent ASFV (Armenia 2007) were analyzed for the levels of ASFV DNA, gDNA (beta-actin and NR6A1) and mtDNA (mtCytb), as well as for the presence of a cytoplasmic enzyme (LDH). An overview of the results obtained is presented in Supplementary Table S1. By reference to standard curves, levels of ASFV DNA, beta-actin DNA and mtCytb DNA were converted from Ct values into genome copy numbers/mL (standard curves used for conversion of Ct values to genome copy numbers are shown in Supplementary Table S2). Genome copy numbers/mL are shown in Figure 1, panels A–C (domestic pigs) and panels E–G (wild boar). LDH activities are shown as mU/mL in the same Figure, panel D (domestic pigs) and panel H (wild boar).

Levels of the beta-actin gene in serum were similar in both domestic pigs and wild boar before inoculation (0 dpi), at around 10^5^ genome copies/mL (mean 2.53 × 10^5^ genome copies/mL), and remained relatively stable until the sampling at 8 dpi (Figure 1B,F). In ASFV-infected domestic pigs and wild boar, the level of the beta-actin gDNA in the sera increased in samples obtained at the end of the infection, i.e., at 8 dpi and postmortem to around 10^7^ genome copies/mL (mean 2.80 × 10^7^ genome copies/mL). At this time (8 dpi and postmortem), levels of ASFV DNA in the blood of the infected pigs were at around 8.59 × 10^8^ genome copies/mL (Figure 1A,E). Specifically, levels of beta-actin gDNA increased between 0 dpi and euthanasia (postmortem) in serum from the three ASFV-infected domestic pigs (pigs no. 2837, 2839 and 2840). This difference of more than 10 cycles (changes in Ct values from 32.9 to 36.1 to Ct values from 22.8 to 22.9, see Supplementary Table S1) represents a greater than 1000-fold increase (2^10^ = 1024-fold) in gDNA levels. A similar level of increase in gDNA in serum was observed for most of the ASFV-infected wild boar sampled prior to, and at the end of, the course of infection (0 dpi and 8 dpi, respectively), namely wild boars no. 2856, 2857 and 2859. In these three wild boar, a difference of 8–10 cycles was observed (changes in Ct values from 32.0 to 33.7 to Ct values from 23.5 to 23.8), i.e., a 256-fold to a 1024-fold increase in cfDNA. One exception was wild boar no. 2858, in which no real increase in gDNA levels in serum occurred between 0 dpi and 8 dpi. This wild boar also had a lower level of ASFV DNA in serum at 8 dpi compared to the other infected animals sampled at the end of the experiment (see Supplementary Table S1).

During the entire course of infection, there was a correlation between the level of ASFV DNA present in serum and the presence of gDNA. Using the Spearman rank correlation coefficient, across all animals at all time points, a correspondence between the presence of ASFV DNA and the beta-actin DNA in serum of r = 0.610 was found, yielding a two tailed *p*-value of <0.0001. No major difference was observed when analyzing domestic pigs and wild boar separately (r = 0.645, *p* < 0.0001 for domestic pigs only, r = 0.647, *p* = 0.0001 for wild boar only).

For mtDNA, a higher initial level was observed in serum from both domestic pigs and wild boar when compared to the levels of actin gDNA. Prior to infection, the mean level was 3.07 × 10^6^ genome copies/mL. This remained relatively stable until the samplings on 8 dpi (Figure 1C,G). At 8 dpi, and postmortem, the mean level of mtDNA was elevated to 6.76 × 10^7^ genome copies/mL. This increase was, however, mainly driven by an increase in the mtCytb levels in domestic pigs. In these animals, levels at 8 dpi and postmortem reached a mean of 9.12 × 10^7^ genome copies/mL (i.e., increased about 30-fold), compared to a mean of 3.22 × 10^7^ genome copies/mL in wild boar sampled at 8 dpi (also see Figure 1C,G), which is little changed. Specifically, levels of mtDNA changed less than the gDNA in the same domestic pigs (pigs no. 2837, 2839 and 2840) and even less in the wild boar (wild boar no. 2856, 2857, 2858, 2859) when levels prior to inoculation were compared to those at the end of the course of the infection. Thus, in the three domestic pigs, a difference of around 5–9 cycles in individual pigs (changes in Ct values from 26.1 to 27.7 to Ct values from 17.5 to 21.3), representing a 32-fold to 512-fold increase in mitochondrial DNA levels, was observed. In the four wild boar, the increase in the level of mitochondrial DNA was even less pronounced, with a difference of around 2–3 cycles (changes in Ct values from 22.5 to 24.4 to Ct values from 19.7 to 21.0), representing 4-fold to 32-fold changes (see Supplementary Table S1).

Using the Spearman rank correlation coefficient, across all animals at all time points, a correlation between the levels of ASFV DNA and the mtCytb DNA in serum of r = 0.567 was found, yielding a two tailed *p*-value of <0.0001. However, when looking at domestic pigs and wild boar separately, this correlation was much stronger for domestic pigs (r = 0.648, *p* < 0.0001), when compared to wild boar (r = 0.383, *p* = 0.0369).

An increase in the levels of the cytoplasmic enzyme LDH was observed in serum from some, but not all, of the ASFV-infected suids (Figure 1D,H). A high background level was observed in one wild boar (no. 2849) at 0 dpi (Figure 1H). Most likely this was due to hemolysis of the blood and red-staining of the serum from this animal (see Supplementary Table S1). Again, using the Spearman rank correlation, no major difference was observed when analyzing domestic pigs and wild boar separately (r = 0.646, *p* < 0.0001 for domestic pigs only, r = 0.657, *p* < 0.0001 for wild boar only). When considering all animals at all time points, the value of r = 0.560, *p* < 0.0001 was determined.

Assays designed to distinguish between the SNP g.299084751 C > T in the suid genomic *NR6A1* gene were able to show large increases in the levels of this genomic DNA in sera from the ASFV-infected suids during the course of the infection (Table 1), consistent with the changes in beta-actin gDNA (see Figure 1B,F).

Domestic pigs are expected to be homozygous for the T allele, while wild boar can be either homozygous for the C allele or heterozygous with both alleles. The latter is demonstrated for two of the wild boar (animals no. 2853 and no. 2854) that tested positive for both the C allele and the T allele on 0 dpi and 5 dpi (Table 1). In ASFV-infected wild boar, elevated levels of the C allele of the *NR6A1* gene were observed in three out of four wild boar at 8 dpi, while the same was observed for the T allele of the gene in three ASFV-infected domestic pigs at 8 dpi and again postmortem.

## 4. Discussion

During infection, with highly virulent variants of the genotype II ASFV, a peracute to acute disease progression is most often observed in infected wild boar and domestic pigs. This presents as a rapid disease progression, with high fever and a range of mostly non-specific clinical signs [9,10,11,12,18,19]. The infection is associated with severe dysregulation of the immune system and cell death [13,14]. Cell death can lead to the release of fragmented cfDNA into the circulation [17], and could thus serve as a marker for acute infection with the currently circulating virulent ASFV variants. We have previously investigated the release of various potential biomarkers for ASFV infection in domestic pigs [18]. Those pigs were infected with a highly virulent genotype II ASFV from Poland. More specifically, we looked into the release of beta-actin gDNA, cytochrome b mtDNA and the cytoplasmic LDH protein into serum obtained from the pigs [18]. In the current study, we investigated the release of the same markers into serum obtained from domestic pigs and also wild boar experimentally infected with a highly virulent genotype II ASFV from Armenia [19].

The levels of ASFV DNA in serum obtained from the infected domestic pigs and wild boar in this study are in accordance with the reported course of infection in the infected animals. In particular, the later detection of ASFV DNA in the infected domestic pigs when compared to wild boar is consistent with the reported shorter incubation period in the infected wild boar (4 days) when compared to the infected domestic pigs (7 dpi) [19]. At the end of the study period, all four infected wild boar reached the humane endpoints, while this was only the case for three out of the four infected domestic pigs [19]. This also corresponds well to ASFV DNA only being present in serum from three out of the four domestic pigs (Figure 1A) but in all four wild boar at 8 dpi (Figure 1D). Detection of ASFV DNA in serum samples in the current study corresponded well with the detection of the viral DNA in EDTA-stabilized blood samples obtained from the same animals on the same sampling days. Viral DNA was detected in EDTA-stabilized blood from wild boar no. 2851 (#51 in the previous study [19]) at 3 dpi and in the same material from three and four wild boar sampled at 5 dpi and 8 dpi, respectively. In domestic pigs, the lack of detection of ASFV DNA in serum obtained at 3 dpi and 5 dpi and the detection of ASFV DNA in only three out of four animals at 8 dpi is also in agreement with the results described for EDTA-blood samples [19].

The large increase (ca. 1000-fold) in the level of gDNA in sera from ASFV-infected domestic pigs and wild boar in this study is consistent with our earlier study, in which a large increase in levels of beta-actin gDNA was observed in sera obtained from ASFV-infected domestic pigs [18]. In the current study, it was shown that levels of gDNA (both beta-actin and NR6A1 DNA) increased in both domestic pigs and wild boar in the late stages of infection with ASFV. Furthermore, using the *NR6A1* gene as a target, it was also demonstrated that this assay clearly distinguished between samples of domestic pig and wild boar origin. The assay targets the SNP g.299084751, which is associated with the number of thoracic and lumbar vertebrae in suids [24,25]. Studies have shown that the majority of domestic pigs are homozygous for the g.299084751 T, while wild boar are heterozygous or homozygous for the wild type allele g.299084751 C [24], which was also observed here.

We have previously shown that levels of mtCytb in serum increased after infection of domestic pigs with virulent ASFV, but to a lesser extent compared to gDNA [18]. This was also observed in the current study. Presumably this could be due to the higher baseline of mtDNA in serum when compared to genomic DNA. It should be noted that, as also previously discussed, the target for the mtDNA sequence in the qPCR is relatively large (274 bp) [22], which could be sub-optimal for the detection of small fragments of cfDNA [16]. For comparison, the target in the beta-actin assay is only 114 bp [20]. Interestingly, mtDNA seems to correlate better with ASFV viral loads in domestic pigs than in wild boar, although baseline levels of mtDNA in the serum from the two types of pigs were rather similar. At the end of the study, infected wild boar had viral DNA levels in serum that were comparable to those of the infected domestic pigs, so differences in viral load do not seem to explain the observed difference. Even though comparable levels of viral DNA were detected in domestic pigs and wild boar, the ASFV-infected wild boar had a more rapid and severe disease progression when compared to the infected domestic pigs. Hence, wild boar developed viremia and subsequently more marked clinical signs and elevated temperatures earlier when compared to domestic pigs. The continued course of infection, e.g., the duration of clinical courses to the humane endpoints, was, however, similar for the two species [19], and differences observed in mtDNA levels can probably not be attributed to differences in the severity of the infection in domestic pigs versus wild boar. A difference in other biomarkers of stress physiology, assessed in saliva and serum, has been shown between wild boar and domestic pigs infected with a highly virulent ASFV [26]. As also mentioned by those authors, further studies exploring the differences in stress responses [26], as well as responses to cellular damage, between domestic pigs and wild boar could be warranted.

In this study, we have demonstrated an increase in the cellular enzyme LDH in serum of both infected domestic pigs and wild boar in the later stages of infection (at 8 dpi and postmortem). The same increase has been demonstrated in domestic pigs following infection with another highly virulent genotype II ASFV [18,27]. As observed previously, the increase was less marked when compared to the change in the level of cfDNA in the infected suids [18]. In a recent study [26], LDH levels in serum have been reported to correlate strongly with the clinical scores and viral genome loads of the virus in domestic pigs and wild boar infected with a highly virulent genotype II ASFV. It should be noted that for the current study we applied a non-parametric statistical test for correlation, while the previous study applied a parametric test following logarithmic transformation of their data in order to obtain a normal distribution [26]. In general, non-parametric tests are more conservative compared to parametric tests.

As previously, the current study underlines that the parallel detection of ASFV DNA and cfDNA, using qPCR, can be a convenient way to follow the course of infection within domestic pigs during pathogenesis studies, and the current study shows that cfDNA could also be a marker of acute infection with ASFV in wild boar. For example, using an ASFV qPCR assay in which swine gDNA, e.g. beta-actin gene, is included as an internal control [20] would allow for the easy and simultaneous detection of ASFV DNA and gDNA levels during such studies.

The findings of this study demonstrate that, using the methods described in the current study, of the analyzed markers, cfDNA seems to be the optimal marker for cell death during infection with highly virulent ASFV in both domestic pigs and wild boar. As the current study and the previously reported studies on biomarkers in pigs were conducted in samples obtained from pigs infected with highly virulent genotype II ASFVs [18,26], future studies using attenuated strains of the genotype II ASFVs could be warranted to investigate the role of cfDNA in the pathogenesis of ASFV.

In conclusion, cfDNA can be a useful marker for cell death during acute infection with highly virulent variants of ASFV in both domestic pigs and wild boar, and release of cfDNA could offer one method to elucidate the pathogenesis of ASFV infections further.

## Figures and Tables

**Table 1:** Detection of NR6A1 gDNA in serum from domestic pigs and wild boar. Nucleic acids isolated from selected serum samples were assayed for the levels of NR6A1 gDNA, for the T allele (DP) and C allele (WB). Results are presented as Ct values. The colored text indicates that ASFV DNA was detected in serum from the pig or wild boar on that sampling day—with the text in red indicating ASFV DNA levels above 10^8^ ASFV DNA genome copies/mL and the text in orange indicating a level of ASFV DNA below 10^8^ genome copies/mL (also see Figure 1A,E).

	SNP T (“DP *”)/SNP C (“WB **”)				
Animal ID	0 dpi ***	3 dpi	5 dpi	8 dpi	PM #
2825 (DP)	33.2/No Ct	##	-	-	-
2826 (DP)	32.7/No Ct	-	-	-	-
2827 (DP)	30.3/No Ct	-	-	-	-
2831 (DP)	-	32.3/No Ct	-	-	-
2832 (DP)	-	31.8/No Ct	-	-	-
2833 (DP)	-	28.5/No Ct	-	-	-
2834 (DP)	-	-	32.0/No Ct	-	-
2835 (DP)	-	-	32.5/No Ct	-	-
2836 (DP)	-	-	32.2/No Ct	-	-
2837 (DP)	35.1/No Ct	-	-	23.3/No Ct	25.7/No Ct
2838 (DP)	31.5/No Ct	-	-	30.4/No Ct	33.2/No Ct
2839 (DP)	31.0/No Ct	-	-	22.8/No Ct	27.9/No Ct
2840 (DP)	31.5/No Ct	-	-	23.6/No Ct	28.5/No Ct
2850 (WB)	-	No Ct/32.2	-	-	-
2851 (WB)	-	No Ct/30.1	-	-	-
2853 (WB)	34.9/33.8	-	31.8/29.3	-	-
2854 (WB)	32.9/31.3	-	33.3/31.4	-	-
2855 (WB)	No Ct/32.6	-	No Ct/30.1	-	-
2856 (WB)	No Ct/29.6	-	-	No Ct/20.8	-
2857 (WB)	No Ct/29.5	-	-	No Ct/20.3	-
2858 (WB)	No Ct/29.2	-	-	No Ct/28.9	-
2859 (WB)	No Ct/30.0	-	-	No Ct/21.1	-

* DP = domestic pig; ** WB = wild boar; *** dpi = days post infection; # PM = postmortem; ## = analysis was not performed.

## Data Availability

The original contributions presented in this study are included in the article/Supplementary Materials. Further inquiries can be directed to the corresponding author.

## Supplementary Materials

The following supporting information can be downloaded at https://www.mdpi.com/article/10.3390/pathogens14121228/s1, Table S1: Overview of data from pigs; Table S2: Standard curves for calculation of genome copy numbers.
